# Assessment of Selected Morphological, Physical and Chemical Parameters of the Teeth of the Offspring of Female Rats Exposed to 2,3,7,8-Tetrachlorodibenzo-*p*-dioxin (TCDD), Taking into Account the Protective Role of Selected Antioxidants—Preliminary Study

**DOI:** 10.3390/ani12040484

**Published:** 2022-02-16

**Authors:** Maciej Dobrzyński, Anna Nikodem, Joanna Klećkowska-Nawrot, Karolina Goździewska-Harłajczuk, Maciej Janeczek, Marzena Styczyńska, Piotr Kuropka

**Affiliations:** 1 Department of Pediatric Dentistry and Preclinical Dentistry, Wroclaw Medical University, Krakowska 26, 50-425 Wroclaw, Poland; 2 Department of Mechanics, Materials and Biomedical Engineering, Faculty of Mechanical Engineering, Wrocław University of Science and Technology, Wybrzeże Wyspiańskiego 27, 50-370 Wroclaw, Poland; anna.nikodem@pwr.edu.pl; 3 Department of Biostructure and Animal Physiology, Division of Animal Anatomy, Faculty of Veterinary Medicine, Wroclaw University of Environmental and Life Sciences, Kożuchowska 1, 51-631 Wroclaw, Poland; joanna.kleckowska-nawrot@upwr.edu.pl (J.K.-N.); maciej.janeczek@upwr.edu.pl (M.J.); 4 Faculty of Biotechnology and Food Sciences, Wroclaw University of Environmental and Life Sciences, Chełmońskiego 37/41, 51-630 Wroclaw, Poland; marzena.styczynska@upwr.edu.pl; 5 Department of Biostructure and Animal Physiology, Division of Histology and Embryology, Faculty of Veterinary Medicine, Wroclaw University of Environmental and Life Sciences, Norwida 25, 50-635 Wroclaw, Poland; piotr.kuropka@upwr.edu.pl

**Keywords:** dental structure, 2,3,7,8-tetrachlorodibenzo-*p*-dioxin (TCDD), AhR receptor, *Buffalo* rat, histology, α-tocopherol, acetylsalicylic acid

## Abstract

**Simple Summary:**

The present study aimed to determine the possibility of limiting potential dioxin disorders of the structure of hard tissues in the offspring of intoxicated rat mothers by simultaneous administration of α-tocopherol (vitamin E) or acetylsalicylic acid (ASA). The levels of magnesium (Mg), calcium (Ca) and phosphorus (P) contained in bone tissue as indicators of the process of mineralization of hard tissues were also determined. The chaps were harvested from the offspring of eight female *Buffalo* rats for testing. Selected morphological, chemical and physical parameters of the teeth of the offspring of female rats from the experimental groups were analyzed. The analysis showed the effect of vitamin E and ASA for the content of Mg, Ca and P. In combination with 2,3,7,8-tetrachlorodibenzo-*p*-dioxin (TCDD), vitamin E and ASA, they positively inhibit the inflammatory process, preventing the leaching of Ca and Mg from the bones. ASA counteracted this phenomenon much more effectively than vitamin E. An increase in the number of dentiform cells was observed, but a slightly lower Ca content, which means that the tooth walls in this group have a lower density. Despite the use of compounds protecting the teeth against the harmful effects of TCDD, disturbances in the structure of the tooth crown cusps were still observed.

**Abstract:**

The studies conducted so far indicate a negative effect of dioxins on the structure of the alveolar bone and teeth, especially in the developmental period in rats. The research aimed to analyze the indirect effect of dioxins contained in the body of female rats on the structure of the dental organ in their offspring in the neonatal period and to determine the possibility of reducing potential dioxin disorders of the structure of hard tissues in the offspring of intoxicated mothers by simultaneous administration of vitamin E or acetylsalicylic acid (ASA). Another goal of the research was to determine the level of magnesium, calcium and phosphorus contained in bone tissue as indicators of the mineralization process of hard tissues in rats, in the case of using 2,3,7,8-tetrachlorodibenzo-*p*-dioxin (TCDD) and acetylsalicylic acid or α-tocopherol. The experiment was carried out on eight female rats of the *Buffalo* strain divided into four groups. From the offspring of eight females, the mandibles were removed from the mandibular joints, and then, after the removal of soft tissues, they were prepared for individual tests. Selected morphological, chemical and physical parameters of the teeth of the offspring of female rats from the experimental groups were analyzed. The analysis showed the effect of vitamin E and ASA on the content of Mg, Ca and P. In combination with TCDD, vitamin E and ASA, they positively inhibit the inflammatory process, preventing the leaching of Ca and Mg from the bones. ASA counteracted this phenomenon much more effectively than vitamin E. Detailed analysis of the tooth morphology showed that the molars’ crowns exhibit shape disturbances under the influence of TCDD. Individual nodules in teeth T1, T2, T3 did not fuse, and the roots showed signs of hypertrophy. The study confirmed the negative effect of TCDD on tooth development. Teeth arising early in development are the most sensitive to the disorders, while the later ones are less exposed to the toxic effects of TCDD transmitted by the mother.

## 1. Introduction

Dioxins are formed in any thermal process and are characterized by the presence of organic matter, chlorine, high temperature and low oxygen concentration. Dioxins are emitted not only during the incineration and disposal of waste but also in various technological processes. Important routes of penetration of dioxins into the body are the digestive system, respiratory tract and skin. Dioxins have a high affinity for fats and can dissolve in them, thanks to which they can be easily supplied and stored in the body’s adipose tissue. Dioxins circulate in various food chains, including their presence in organisms living in water [1]. The most toxic and model form of dioxins is TCDD (2,3,7,8-tetrachlorodibenzo-*p*-dioxin). Based on the results of experimental studies carried out in the Wrocław Center, it has been shown that 2,3,7,8-tetrachlorodibenzo-*p*-dioxin (TCDD) causes, by inducing inflammation, the simultaneous destruction of connective tissue [2,3]. Additionally, TCDD inhibits the synthesis of collagen I and induces oxidative stress increasing the concentration of pro-inflammatory interleukins activating osteoclastogenesis [3,4,5]. The studies of other authors show that inhibition of alkaline phosphatase activity and some mineralization initiators by dioxin may affect the formation of less mineralized tissues [4,5,6].

Therefore, it was assumed that dioxin may affect the bone structure of the alveolar process and teeth, especially in the developmental period. This seems to be confirmed by the observed changes in the external appearance of the offspring of rats administered TCDD [7,8]. The clear underdevelopment of the skeleton found in the above studies may suggest the existence of developmental disorders in the field of hard tissues. Dioxins, also by influencing the activity of certain hormones (estrogens, corticosterone, T3), can modify the proper development of hard tissues. By disrupting the estrogenic balance, TCDD contributes to the reduction in calcium deposition in mineralized tissues, and by increasing the concentration of corticosterone, it stimulates the breakdown of collagen fibers. Finally, by influencing the concentration of the active form of vitamin D3, dioxin contributes to the inhibition of fibroblast activity [7,8,9]. This was confirmed by our own studies; however, it seems to have a higher importance in young animals than in old ones. The activation of the aryl hydrocarbon receptor (AhR) plays a key role in the pathomechanism of TCDD activity, only one of the consequences of which is the disturbance in the synthesis of collagen fibers [4,10], which may result in disturbances in the spatial structure of bone tissue and hard tissues of teeth (enamel, dentin and cement). Therefore, it seems right to look for pharmacological agents that can bind to the AhR receptor, thus preventing dioxin from making active connections with it. As shown by recent experimental studies, two well-known drugs: tocopherol and acetylsalicylic acid, are characterized by antagonistic properties toward the AhR receptor [5,11,12], which raises the prospect of a potential extension of indications for their use. An additional protective property of the above substances is the ability to inhibit the inflammatory reaction caused by dioxins [4,10,11,12].

The rat is a commonly used mammal for odontogenesis studies. The molars of these mammals are very similar in terms of odontogenesis, physiology and histological structure to human milk teeth [13,14]. The main difference between tooth development in rats and humans is that in rats, the odontogenesis process is a monophyodontal process [14,15,16,17]. Moreover, a characteristic feature of rat incisors is the fact that their growth, mineralization, eruption and abrasion are constant processes lasting throughout the life of the animal [14,15,16,17].

Assessment of the impact of specific metabolic changes in the body on the odontogenesis process should be performed in very young animals. In rats, the odontogenesis process ends around the 35th day of individual life, after which catabolic processes begin to dominate over anabolic processes and may disturb the proper interpretation of even subtle consequences of developmental conditions, making it unreliable or even impossible [17,18,19].

Taking all of this under consideration, the aim of this study was to compare tooth malformation in rat offspring whose mothers were intoxicated by TCDD and possible prevention by known antioxidants such as α-tocopherol and acetylsalicylic acid using the micro CT technique with histological analysis.

## 2. Materials and Methods

### 2.1. Animals and Procedure

Eight female rats of the *Buffalo* strain (age 9–11 weeks, weight 130–150 grams) were used in the experiment. The animals were housed in polystyrene cages with "Labofeedh" food and water.

The females were randomized into 4 groups, each consisting of 2 animals:

I. A control group of females (C), not treated with any chemicals;
II. A group of females (TCDD) who received a solution of 2,3,7,8-tetrachlorodibenzo-*p*-dioxin (TCDD) in a single dose of 5 μg/kg BW them;
III. A group of females (TCDD + E) administered a solution of 2,3,7,8-tetrachlorodibenzo-*p*-dioxin (TCDD) in a single dose of 5 μg/kg BW them. and a solution of α-tocopherol acetate at the dose of 30 mg/kg BW/day *s.c.* was administered for a period of 3 weeks;
IV. A group of females (TCDD + ASA) administered a solution of 2,3,7,8-tetrachlorodibenzo-*p*-dioxin (TCDD) in a single dose of 5 μg/kg BW them and administration of acetylsalicylic acid suspension in starch solution at a dose of 50 mg/kg BW/day *per os (p.o.)* for a period of 3 weeks.

After a period of 3 weeks after administration of TCDD in individual groups (TCDD, TCDD + E, TCDD + ASA), the above-mentioned groups of females and group C were matched with randomly selected males from the same strain, not treated with any chemical substances. After the mating period, pregnant females were placed in separate cages.

TCDD females’ deliveries were extended over 3 weeks compared to the control group in which deliveries occurred within 4 days. The number of offspring born to females in individual groups was different:

1. Newborns (NC)—10 individuals (from C females);
2. Newborns (NTCDD)—4 individuals (from TCDD females);
3. Newborns (NTCDD + E)—7 individuals (from TCDD + E females);
4. Newborns (NTCDD + ASA)—6 individuals (from TCDD + ASA females).

All newborns were kept in cages for another 30 days, after which they were sacrificed, and the material was collected for testing (animals underwent barbiturane anesthesia; thiopental (Biochemie GmbH, Kundl, Austria) was injected intraperitoneally at a dose of 120 mg/kg of BW).

The mandibles were dissected from the temporomandibular joints, and then, after the removal of soft tissues, they were prepared for individual examinations.

Pharmacological agents and chemicals used in the experiment:

1. α-tocopherol acetate (an oil solution of the drug prepared on an individual order by Hasco-Lek S.A. in Wroclaw) (vitamin E);
2. Acetylsalicylic acid—Aspirin (Bayer) (drug suspension in starch solution was prepared at the Department of Medical Biochemistry, Wroclaw Medical University);
3. Thiopental (Biochemie GmbH, Kundl, Austria);
4. Standard solution of 2,3,7,8-tetrachlorodibenzo-*p*-dioxin (TCDD) (Greyhound Chromatography and Allied Chemicals, cat. at the Department of Organic Technology, Wroclaw University of Technology);

The research material consisted of 16 prepared fragments of the mandibles with incisors and molars.

### 2.2. Methods

The collected mandibles were divided into two from each individual, and one-half underwent the microCT and chemical spectroscopic analysis (determination of calcium, magnesium and phosphorus content), and the second half underwent the histological analysis. The purpose of microCT analysis was to measure the density and geometric parameters of the tissues of rats’ teeth.

#### 2.2.1. Histological Research

The mandibles of rats immediately after extraction were fixed in a 4% formalin solution, pH 7.2–7.4 for 48 hours, then rinsed in running water for 24 hours. After rinsing, the material was decalcified in a mixture of concentrated acids: hydrochloric and formic (for 24 hours), dehydrated in an alcohol series and embedded in paraffin. The blocks were cut in the midplane of the tooth, longitudinally to the mandible edge into sections 7–9 μm thick and then dewaxed in xylene and stained with hematoxylin and eosin according to Delafield. The slides were observed using a Nikon Eclipse 80i (Tokyo, Japan) optical microscope in transmitting light.

#### 2.2.2. Determination of the Level of Ca, Mg and P

##### Mineralization of Research Material

The samples were "wet" mineralized in a closed microwave system. A total of 5 cm^3^ of concentrated nitric acid (V) p.a. was added to an aliquot of a homogeneous sample (from 0.1 to 0.5 g) and 1 cm^3^ of concentrated hydrogen peroxide p.a., then the samples were mineralized in the microwave MARS 5 (CEM, USA) sample preparation system. The minerals were quantitatively transferred to 10 cm^3^ measuring vessels with redistilled water. The mineralization was carried out by the Polish Standard PN-EN 13805: 2003 "Foodstuffs. Determination of trace elements. Pressure mineralization" [20].

##### Determination of Elements in the Research Material

‑ *Determination of calcium content by atomic emission spectrometry:*

Determination of the calcium content in an acetylene/air flame was carried out by atomic emission spectrometry using a SpectraAA atomic absorption spectrometer Cal-L Enterprises (Chatsworth, CA, USA) with a Varian AA240FS flame attachment.

The Ca content was determined by the Research Procedure PB-01/AAS developed and used in the Research Laboratory for Atomic Absorption Spectrometry at the University of Life Sciences in Wroclaw.

‑ *Determination of magnesium content by atomic absorption spectrometry:*

Determination of the magnesium content in an acetylene/air flame was carried out by atomic absorption spectrometry using a SpectraAA atomic absorption spectrometer (Chatsworth, CA, USA) with a Varian AA240FS flame attachment.

The determination of Mg content was performed by the Research Procedure PB-02/AAS developed and used in the Research Laboratory for Atomic Absorption Spectrometry at the University of Life Sciences in Wroclaw.

‑ *Determination of phosphorus content by atomic emission spectrometry:*

The phosphorus content of the acetylene/air flame was determined by atomic emission spectrometry using a SpectraAA atomic absorption spectrometer (Chatsworth, CA, USA) with a Varian AA240FS flame attachment.

The P content was determined by the Research Procedure PB-03/AAS developed and used in the Research Laboratory for Atomic Absorption Spectrometry at the University of Life Sciences in Wroclaw.

#### 2.2.3. Measurement of Geometric Parameters and Tissue Density of Rats

Measurements of the density and geometrical parameters of individual teeth were carried out with the use of CTAn and DataViewer programs on reconstructions obtained with the use of the 1172 SkyScan, Bruker® (Kontich, Belgium) computer microtomography. Each of the preparations was recorded with a resolution of 9 µm, with the lamp parameters: 80 kV/124 mA, Al filter. (0.5 mm), with a unit rotation angle of 0.4 degrees. The analysis occurred in the Department of Mechanics, Materials and Biomedical Engineering, Faculty of Mechanical Engineering, Wroclaw University of Science and Technology.

#### 2.2.4. Statistical Analysis

The research results were based on the statistical analysis of the measurement results, which consisted of:

‑ Estimating descriptive statistics (mean, standard deviation);
‑ Checking the normality of the distribution of features using the Shapiro–Wilk test;
‑ Verification of hypotheses about the equality of the level of measurable features with a normal distribution in more than two groups using the analysis of variance (ANOVA).

All hypotheses were verified at the significance level of *p* < 0.05.

The statistical analysis was carried out using the statistical software package Prism 9.3.1, GraphPad Software (San Diego, CA, USA). 

## 3. Results

### 3.1. Histological Research

**Figure 1:**
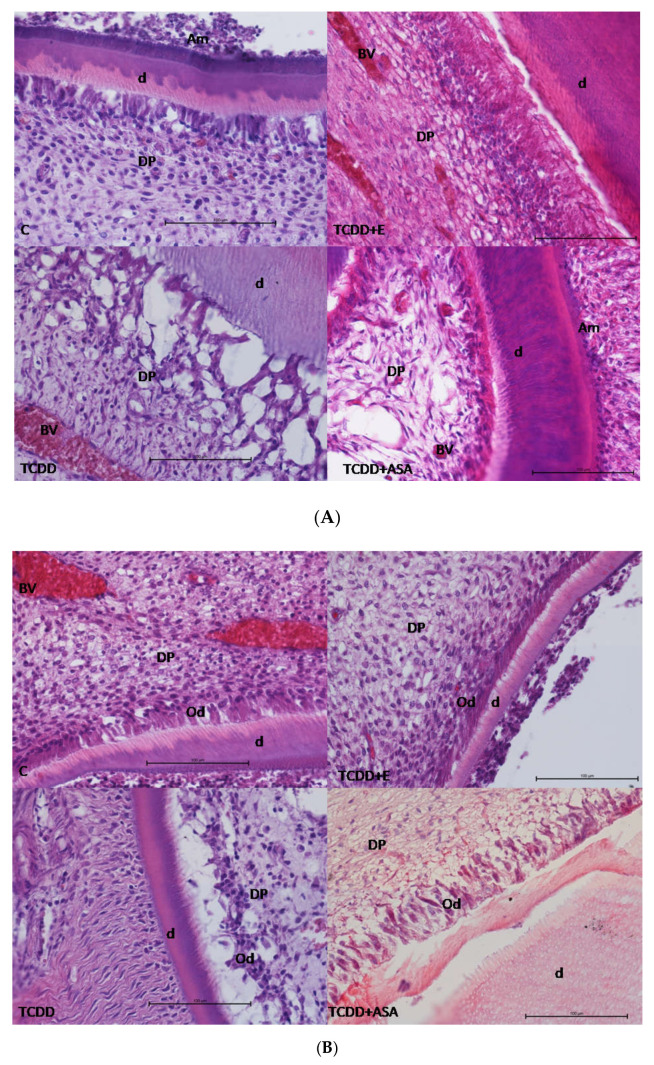
**(A)** Histological structure of the rat molars crown. In C, TCDD + E and TCDD + ASA, well-formed dentine with numerous odontoblasts is noted with a small amount of ameloblasts synthesizing enamel covering the dentin. In the case of the TCDD group, the odontoblasts altered structure. The dentin is not compact and seems to have enlarged dental canaliculi. The blood vessels in all experimental groups are enlarged and contain erythrocytes. d—dentin, DP—tooth pulp, BV—blood vessel, Am—ameloblasts, Od—odontoblasts. (**B**) Histological structure of rat molars roots. Enlarged blood vessels in the control group were also observed in other groups. Detachement and decreased number of odontoblasts in the TCDD group. C: control group; TCDD: 2,3,7,8-tetrachlorodibenzo-*p*-dioxin; ASA: acetylsalicylic acid; E: vitamin E.

Histological examination revealed a decrease in the number of odontoblasts and ameloblasts in both the root formation zone and the dental body in the TCDD group compared to the control groups (Figure 1A,B). This resulted in a decrease in both the thickness of the enamel and the dentin in the crown and in the pulp area. In the pulp, enlargement of the tooth cavity, especially in incisors, and increased number of white blood cells (mainly macrophages and lymphocytes) were noted. In the group in which tocopherol was administered protectively with α-tocopherol, no decrease in the number of odontoblasts or ameloblasts was observed, and ameloblasts, especially within the root area of incisors, were noticeably higher. The expanded blood vessels were noted in crown and roots in all experimental groups in both molars and incisors, whereas in the control group in root area only. The wall thickness was decreased in all TCDD, TCDD + E and TCDD + ASA; however, the numerous odontoblasts in TCDD + E and TCDD + ASA indicate active dentin synthesis (Figure 1B).

### 3.2. Elements’ Composition Analysis

**Figure 2:**
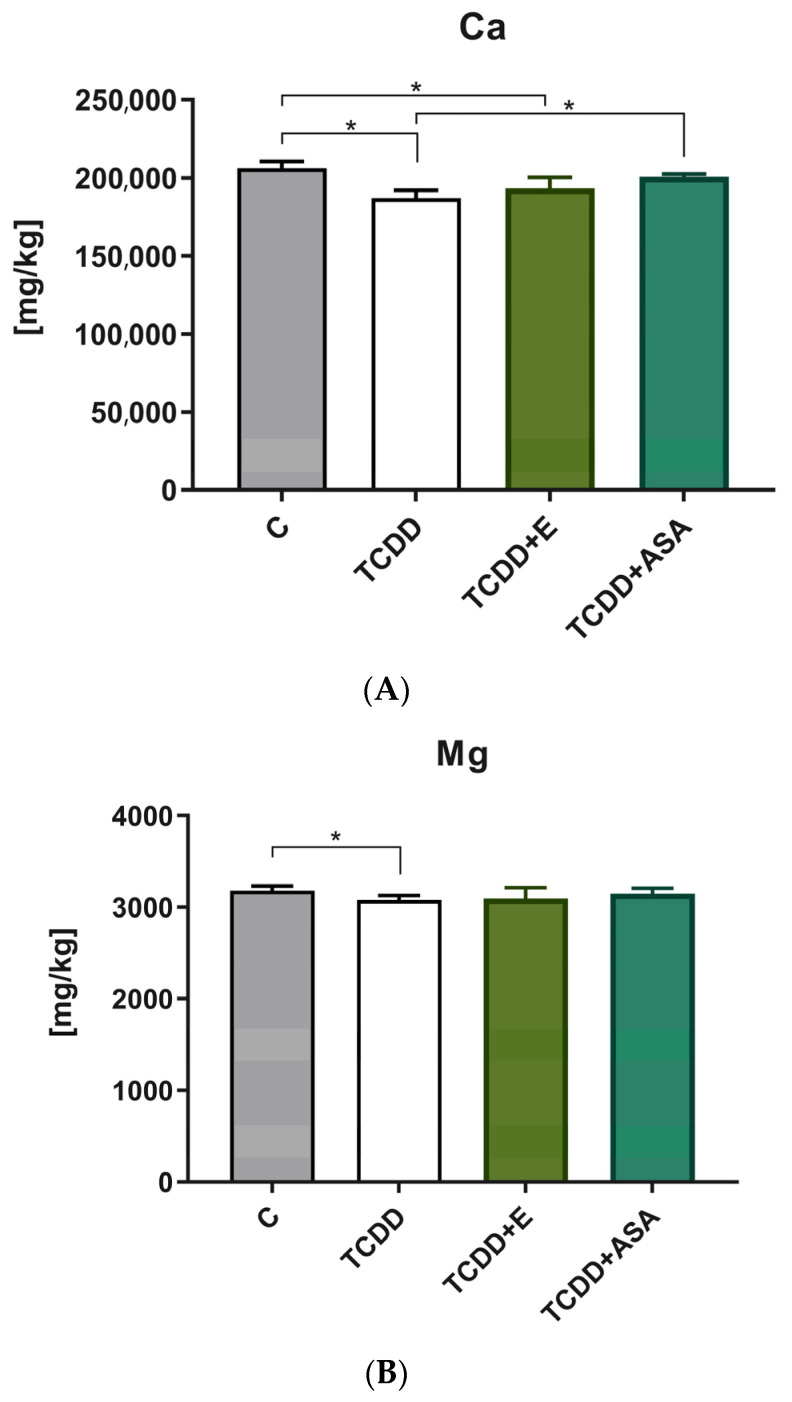

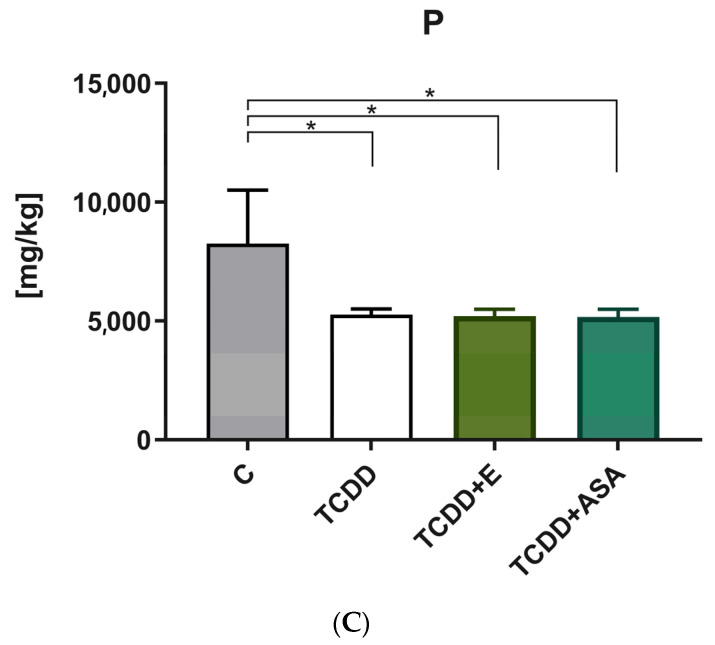
Comparison of the mean and standard deviation values of individual content of (**A**) Ca elements, (**B**) Mg elements, (**C**) P elements. C: control group; TCDD: 2,3,7,8-tetrachlorodibenzo-*p*-dioxin; ASA: acetylsalicylic acid; E: vitamin E, * p < 0.05.

The analysis showed the effect of tocopherol and acetylsalicylic acid on the content of calcium and magnesium (Figure 2A,B). This phenomenon was not demonstrated for phosphorus (Figure 2C). In conjunction with TCDD, vitamin E and ASA positively inhibit the inflammatory process, preventing the leaching of calcium and magnesium from the bones. The content of phosphorus, regardless of the pharmacological agent used, did not change the disturbances in phosphorus content in tooth buds caused by TCDD. It is noteworthy that ASA counteracted this phenomenon much more effectively than vitamin E.

### 3.3. Measurement of Geometric Parameters and Tissue Density of Rats with Histological Picture

**Figure 3:**
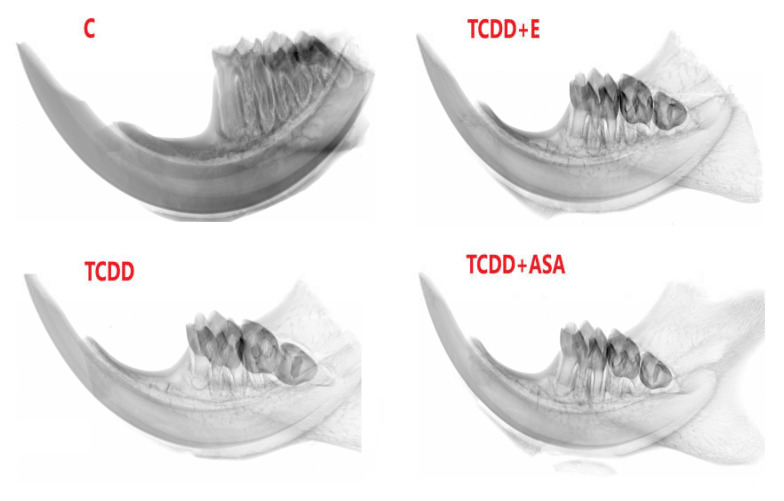
Comparison of topograms of rats’ jaws with incisors and premolars of individual measurement groups: C, TCDD, TCDD + vitamin E and TCDD + ASA. C: control group; TCDD: 2,3,7,8-tetrachlorodibenzo-*p*-dioxin; ASA: acetylsalicylic acid.

As a result of the action of TCDD in the pre- and postnatal period, the odontogenesis of the tooth crown and roots is disturbed. This effect persists despite the protective effect of vitamin E and ASA. Visible thinning and change of incisor geometry in TCDD and its clear improvement in group E. Moreover, a significant enlargement of the tooth cavity is observed in the TCDD group (Figure 3).

**Figure 4:**
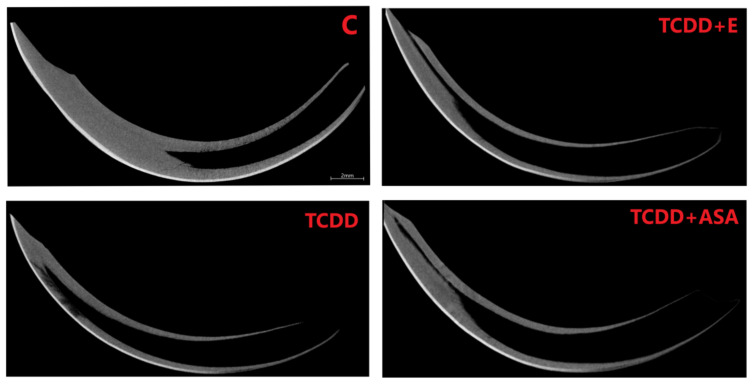
Comparison of the curvature and the wall thickness of the incisors of jaws from individual measurement groups: C, TCDD, TCDD + vitamin E and TCDD + ASA. The increased volume of incisors cavity is noted in all groups where TCDD was used. C: control group; TCDD: 2,3,7,8-tetrachlorodibenzo-*p*-dioxin; ASA: acetylsalicylic acid.

**Figure 5:**
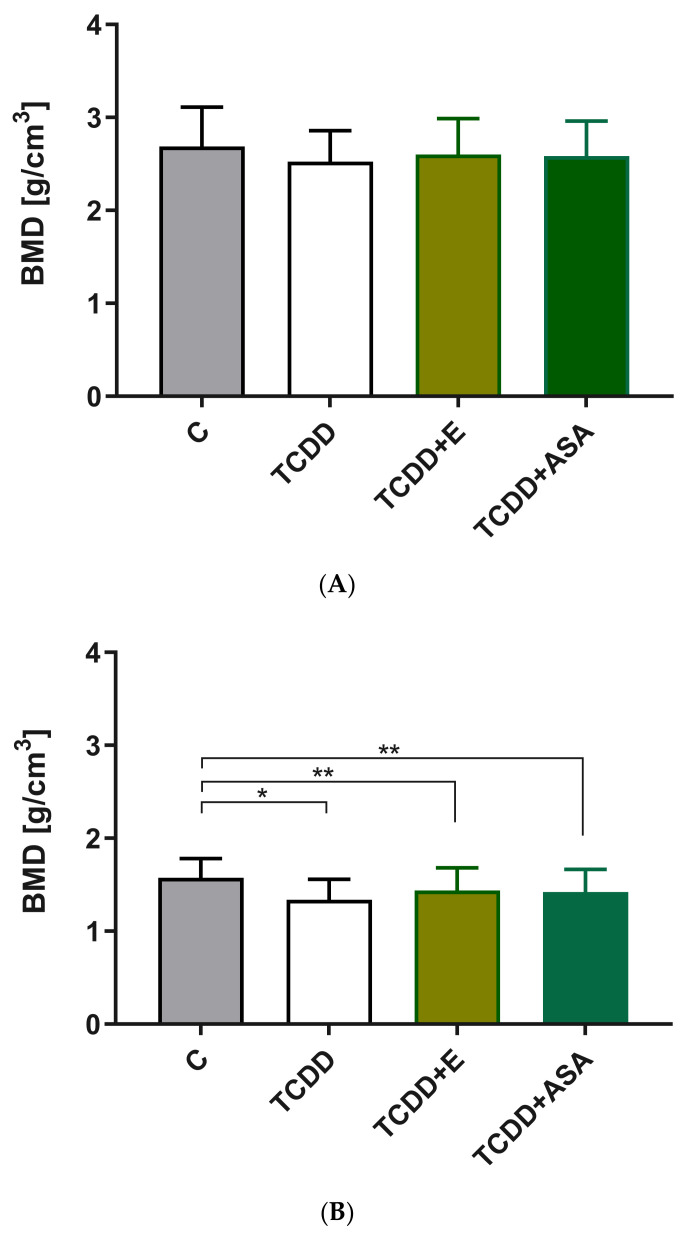
Comparison of mean and SD values of bone mineral density (BMD) determined for (**A**) Enamel tissue in incisors and (**B**) Incisor dentin. * p < 0.05; ** p < 0.1. C: control group; TCDD: 2,3,7,8-tetrachlorodibenzo-*p*-dioxin; ASA: acetylsalicylic acid; E: vitamin E.

In incisors, a change in the form of flattening of the arch geometry is observed in the teeth of the experimental groups (Figure 4). Teeth from these groups have an enlarged tooth cavity, which is filled by the pulp, and there are numerous odontoblasts at its border. Ameloblasts are arranged on the convex surface of the tooth, and their number is the lowest in the TCDD group. Detailed analysis of the tooth morphology showed that the molars’ crowns exhibit shape disturbance under the influence of TCDD (Figure 5). Individual nodules in teeth T1, T2, T3 do not fuse, and the roots show signs of hypertrophy. In the vitamin E and ASA groups, the nodules showed a developmental delay compared to the slightly more advanced control group in the ASA group. Root hypertrophy persists in T2 teeth.

**Figure 6:**
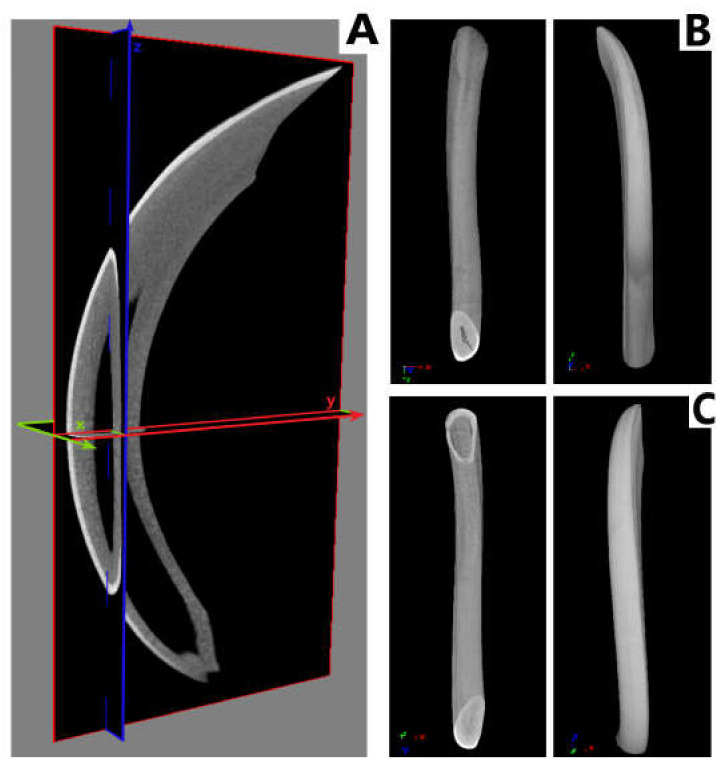
Examples of sections in planes—(**A**) transaxial (XY); coronary (XZ) and sagittal (YZ); (**B**) comparison of an exemplary incisor from the control group; (**C**) comparison of an exemplary incisor of the TCDD + ASA group. TCDD: 2,3,7,8-tetrachlorodibenzo-*p*-dioxin; ASA: acetylsalicylic acid.

For each of the incisors, cross-sections were prepared for the transaxial (XY) and frontal coronary (XZ) planes, for which the values of the orientation angle of the examined cross-section (T.Or (°)) were determined (Figure 6). Teeth torsion was determined as the value of the difference between the maximum and minimum rotation angle (CtAn®, Bruker®, Kontich, Belgium), determined for a particular cross-section along the entire length of the incisor. On the basis of the results presented in Table 1, it can be observed that, in plane XY, the incisor from group C (25.35°) while the lowest for the TCDD group. The biggest difference we can observe in the frontal section, where the torsional value for incisor from group C is 14.6°, whereas, for all groups that received TCDD, the T.Or value was more than twice as high (Table 1).

**Figure 7:**
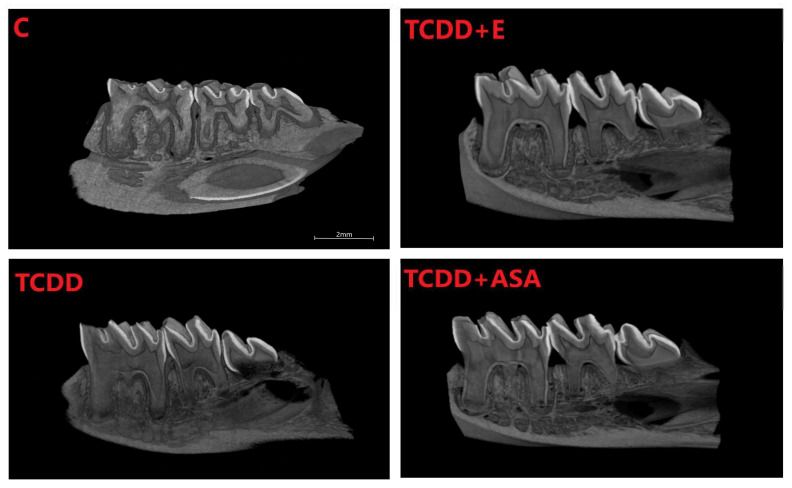
Comparison of the cross-sections of the molars of rats from individual measurement groups: C, TCDD, TCDD + vitamin E and TCDD + ASA. C: control group; TCDD: 2,3,7,8-tetrachlorodibenzo-*p*-dioxin; ASA: acetylsalicylic acid; E: vitamin E.

**Figure 8:**
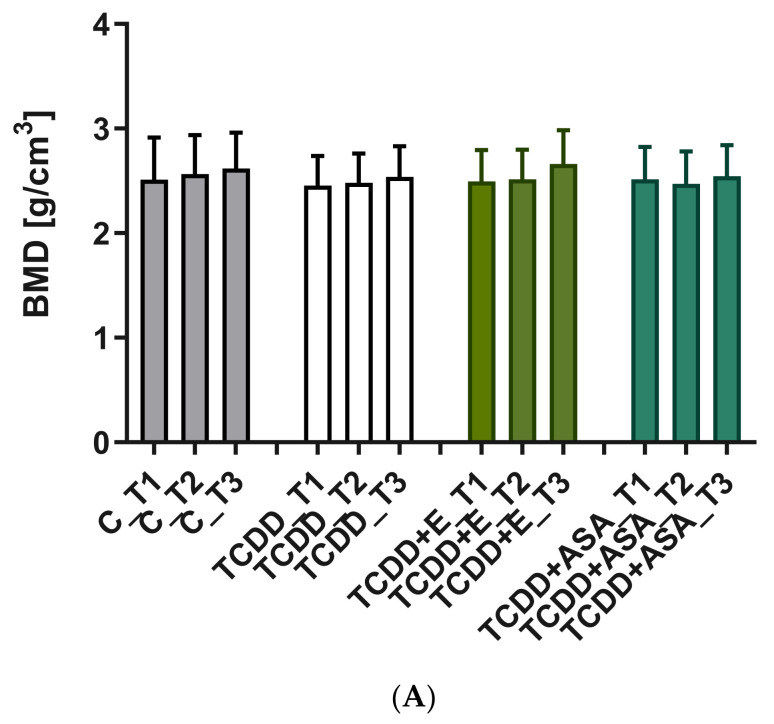

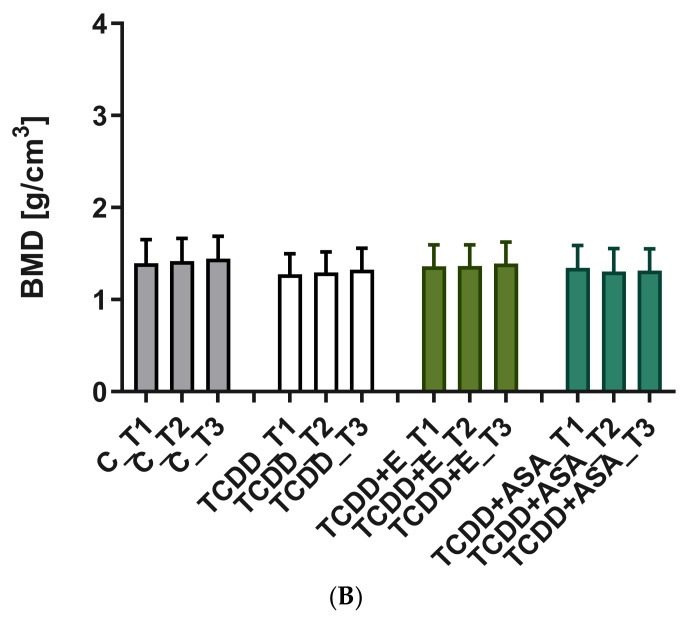
Comparison of the mean and SD values of bone mineral density (BMD) for individual molars T1–T3, determined for (**A**) enamel and (**B**) dentin tissue. C: control group; TCDD: 2,3,7,8-tetrachlorodibenzo-*p*-dioxin; ASA: acetylsalicylic acid; E: vitamin E.

**Figure 9:**
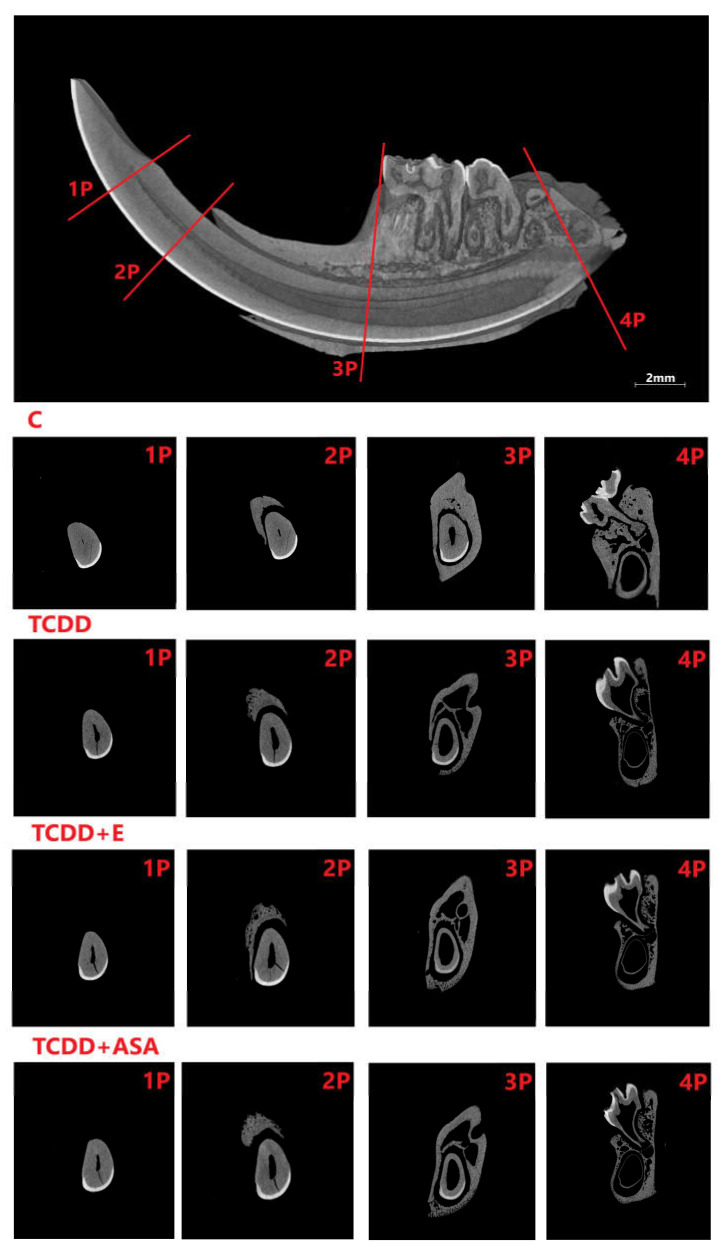
Comparison of the cross-sections of the shafts’ jaws at points 1P–4P of individual measurement groups: C, TCDD, TCDD + vitamin E and TCDD + ASA. C: control group; TCDD: 2,3,7,8-tetrachlorodibenzo-*p*-dioxin; ASA: acetylsalicylic acid; E: vitamin E.

The mineral density measurement was carried out using density phantoms with known values of 0.25 and 0.75 g/cm^3^, which together with the individual preparations were scanned in an 1172 SkyScan, Bruker® (Kontich, Belgium) computer microtomography. The measurement of the density of individual teeth, i.e., incisors and cheek teeth, is shown in Figure 4, Figure 5, Figure 7 and Figure 8. Based on the obtained results, a decrease in the values of the mineral density of tooth tissues (especially for dentin) can be observed after the application of TCDD. There is also a very visible change in enamel thickness (Table 2, Figure 9) in this group, but also changes in the structure of bone tissue (pronounced thinning) at the site of molars (Figure 7).

In this evaluation, the highest disturbances were observed within M3, which in the TCDD group is composed of the crown only with a visible decrease in bone density beneath the roots. In all TCDD-treated groups, enlargement of incisors cavity in the mandible is observed. In addition, teeth nodules on M1, M2 and M3 do not show abrasion, which means disturbance of their growth.

## 4. Discussion

Understanding the course of the odontogenesis process, the biological mechanisms controlling it, and the assessment of the influence of certain innate and acquired factors on this process are possible thanks to experimental studies on animals [13].

The most important features of incisor odontogenesis are [14,15,16,17,18,19]: 1. commencement of the process around the 14th day of fetal life; 2. differentiation of the cells of the enamel-forming epithelium and the dental papilla toward ameloblasts and odontoblasts occurring on the 18–19th day of fetal life; 3. deposition of the first layers of dentin on days 20–21 of utero and enamel several hours later. Rat incisors do not have a developed root but an elongated crown embedded in the socket; they erupt into the oral cavity around the eighth day of individual life. Their growth is constant and even, and about 150 days before the death of the individual, it is significantly slower. The cycle of growth and abrasion takes about 60 days. The average daily increase in the upper incisors is 2.1 mm, while the lower ones are 2.8 mm. The labial surfaces of the upper incisors and the lingual surfaces of the lower teeth are covered with enamel, while the remaining surfaces are covered with softer cement, thanks to which there is characteristic wear of these teeth, which take the shape of a chisel [18,19].

There are four phases in the process of rat molar odontogenesis [14,15,16,17,18,19]. In the first phase, growth, which begins on the 13th day of fetal life, the following processes take place: initiation and multiplication of epithelial cells of the enamel-forming organ. At the same time, cells of the adjacent connective tissue proliferate and form a dental papilla. Around 20 days of gestation, the epithelial cells of the enamel-forming organ differentiate into ameloblasts, while the cells of the dental papilla differentiate into odontoblasts. Between 20 and 21 days of gestation, organic glandular matrix, dentin, cement and alveolar bone are deposited. In the second phase of odontogenesis, as a result of physicochemical processes, the organic core of hard tissues becomes saturated with calcium salts. The process of mineralization of tooth buds takes place with the formation of calculo spheres. The daily rhythm of the growth of hard tissues at the tops of the nodules is 16 μm, while in the area of the roots is 2 μm. In the third phase, on the 19th day of the animal’s life, the eruption of the molar begins, which on the 25th day reaches a functional occlusion. The fourth is the final phase of odontogenesis.

Several factors can disturb tooth formation [4,5,6,7,8,9,10,11,12,13,14,15,16,17,18,19,20,21,22,23,24,25,26,27,28,29,30,31,32]. They include biological (genetic, developmental, viruses, bacteria, cancer), physical or chemical factors. TCDD accumulated in adipose tissue has an influence on the induction of inflammation in the body, even in the third generation. As shown by the works of Dobrzyński et al. [22,23,24,25,26,27], TCDD affects the expression of the Ah receptor (aryl hydrocarbon receptor) in devolving tooth odontoblasts. This receptor, as it has already been mentioned, is closely related to the induction of the inflammatory process on the one hand and, on the other hand, involved in the development and shaping of the tooth. The other authors also confirmed a negative role of TCDD in tooth development [32,33,34,35,36,37,38,39,40]. Our research [24] has shown that TCDD has an influence on the formation of both the tooth crown and its roots and causes changes within the tooth pulp. The influence on odontoblasts and ameloblasts causes the resulting teeth to have thinner walls, the tooth chamber (especially in molars) is enlarged and the shape of the tooth, especially the tooth crown cusps, has an abnormal shape. This has a direct impact on the potential strength of the teeth and their susceptibility to abrasion. It is interesting that the administration of ASA reduces the intensity of infiltration of cells in the lymphatic system but does not show any protective effect on the number of odontoblasts and ameloblasts. Nevertheless, the tooth walls show a slightly increased density both in the number of collagen fibers and the elements contained in them. Tocopherol has a different effect. Here, an increase in the number of tooth-forming cells was observed, while a slightly lower calcium content, which makes the tooth walls in this group less dense. Despite the use of compounds protecting the teeth against the harmful effects of TCDD, disturbances in the structure of the tooth crown cusps were still observed. Interestingly, TCDD and the protective ASA and tocopherol show different effects depending on the position of the tooth in the mandible. In the MCT study, the structure of molars in the mandible was analyzed. From T1–T3 (T-molar), a decrease in the intensity of negative changes in the teeth in the individual groups was observed toward the angle of the mandible, which means that the teeth that develop later are less affected by the toxic TCDD.

The observed changes are the more important because TCDD can accumulate in the environment and be passed down from generation to generation. This will result in an increase in tooth development disorders in future generations unless the increase in the number of dioxins in the environment is significantly slowed down or even stopped completely. The possibilities of counteracting this effect may be largely limited by the use of antioxidants, but they do not reduce the amount of dioxins in the environment. This requires, however, further research.

## 5. Conclusions

The study confirmed the negative effect of TCDD on tooth development. Developmental disorders are primarily molars and incisors, in which there is a decrease in the number of odontoblasts and ameloblasts, and thus the thickness of dentin and enamel. Teeth arising early in development are the most sensitive to the disorders, while the later ones are less exposed to the toxic effects of TCDD transmitted by the mother.

## Figures and Tables

**Table 1:** Comparison of the torsional angles of the tested cross-section (T.Or) for individual measurement groups, according to Figure 6.

	Transaxial Plane (XY)	Coronary Plane (XZ)
T.Or (°)	difference	difference
C	25.354	14.606
TCDD	21.553	30.748
TCDD+E	24.225	33.941
TCDD+ASA	21.879	30.008

C: control group; TCDD: 2,3,7,8-tetrachlorodibenzo-*p*-dioxin; ASA: acetylsalicylic acid; E: vitamin E.

**Table 2:** Comparison of the average values and SD of enamel geometric parameters.

	E.Th (mm)			
Group	IN	T1	T2	T3
C	0.150 ± 0.04	0.103 ± 0.02	0.118 ± 0.02	0.127 ± 0.02
TCDD	0.092 ± 0.02	0.106 ± 0.02	0.115 ± 0.02	0.120 ± 0.03
TCDD+E	0.099 ± 0.03	0.103 ± 0.02	0.112 ± 0.03	0.121 ± 0.03
TCDD+ASA	0.099 ± 0.02	0.101 ± 0.02	0.110 ± 0.02	0.115 ± 0.03
	**EV/TV (%)**			
**Group**	**IN**	**T1**	**T2**	**T3**
C	15.074 ± 2.2	25.523 ± 4.1	27.561 ± 5.1 ^a^	28.851 ± 4.3 ^b^
TCDD	14.030 ± 1.5	28.718 ± 3.4	35.331 ± 4.2 ^a^	44.009 ± 5.2 ^b^
TCDD+E	13.686 ± 2.2	30.031 ± 4.5	35.966 ± 5.3	43.808 ± 5.5
TCDD+ASA	12.506 ± 2.1	28.836 ± 4.8	33.139 ± 5.6	43.095 ± 5.3

(E.Th—enamel thickness and EV/TV—enamel volume fraction in the tooth) for individual incisors (IN) and molars (T1–T3) of the measurement groups, ^a,b^—*p* < 0.05. C: control group; TCDD: 2,3,7,8-tetrachlorodibenzo-*p*-dioxin; ASA: acetylsalicylic acid; E: vitamin E.

## Data Availability

The data presented in this study are available without restriction.
